# Inhibitory effects of propyl paraben, citral, and gallic acid on the growth, patulin biosynthesis, and patulin biosynthetic gene expression of *Penicillium paneum* OM1

**DOI:** 10.3389/fmicb.2026.1949526

**Published:** 2026-09-28

**Authors:** Ji-Yeon Koo, Sung-Yong Hong, Ae-Son Om

**Affiliations:** Department of Food and Nutrition, Hanyang University, Seoul, Republic of Korea

**Keywords:** citral, gallic acid, gene expression, patulin, *Penicillium paneum* OM1, propyl paraben

## Abstract

*Penicillium paneum* is a phytopathogen that can cause fruit decay and produce patulin in pears. Much information is not available about inhibitory effects of food-grade natural compounds on the patulin production by *P. paneum* and its growth. In this study, we evaluated the effects of propyl paraben, citral, and gallic acid on patulin accumulation by *P. paneum* OM1 and its growth on YES (patulin supportive media). Our results showed that its patulin production was below the detection limit due to suppression by 25 μg/mL of propyl paraben or 400 μg/mL of citral and that its growth was inhibited by 14.5% and 42.8%, respectively, at the same levels of each compound. However, gallic acid did not affect the growth of the fungal strain in the range of 100–500 μg/mL, but suppressed the patulin production by 32.9%–37.8% after 7 days of incubation at 25 °C. Additionally, we assessed the expression of 5 key patulin biosynthetic genes in *P. paneum* OM1 under treatment with each compound. It exhibited that the expression of *patK*, *patL*, *patN*, *patG*, and *patE* was down-regulated in *P. paneum* OM1 except for *patE* under gallic acid supplementation. Moreover, when 0.25 μg of propyl paraben was applied onto a half of a pear, the patulin produced by *P. paneum* OM1 was not detected and the application reduced the lesion area on it by 57%. Our data demonstrated the strong inhibitory effect of the low level of propyl paraben on growth of *P. paneum* OM1 and its patulin production in pear. Our findings could provide new insights in utilization of food-grade natural compounds as antifungal and anti-mycotoxigenic agents to prevent patulin contamination of pears by *P. paneum* OM1.

## 1 Introduction

*Penicillium paneum* is a plant pathogen that can cause blue mold decay on fruits including pears (Zhao et al., 2024). It has been reported that some strains of this fungus can also produce patulin in pome fruits and cereal grains, which is a mycotoxin with neurotoxic, genotoxic, teratogenic, and carcinogenic effects on humans (Nielsen et al., 2006; O’Brien et al., 2006; Tannous et al., 2018). It has been proposed that the toxicity of patulin is attributed to formation of adducts with cellular components containing thiol-groups such as glutathione and proteins containing cysteine, and increased generation of reactive oxygen species (ROS) inside cells, leading to inhibition of protein synthesis and transcription, oxidative stress, and cell damage (Mahato et al., 2021; Vidal et al., 2019). Thus, it is necessary to prevent patulin contamination of pome fruits during postharvest storage.

Traditionally, synthetic compounds have mainly been used as antifungal agents for the control of postharvest pathogenic fungi (Neri et al., 2006; Tannous et al., 2018; Zheng et al., 2021). However, because of the occurrence of fungicide-resistant pathogens, the toxicities of the chemical residues on fruits, and environmental concerns, eco-friendly and non-toxic natural antifungal and anti-mycotoxigenic compounds have recently emerged as more effective alternatives to synthetic fungicides (Martinez, 2012; Sanzani et al., 2009; Tannous et al., 2018; Zheng et al., 2021). Plant-based natural products are increasingly gaining more attention for managing mycotoxin contamination of fruits and vegetables during postharvest storage (Tripathi and Dubey, 2004).

A number of previous studies have reported several plant-based natural compounds that suppress mycotoxin production by toxigenic fungi and their growth (Etcheverry et al., 2002; Ferreira et al., 2013; Gowda et al., 2004; Salas et al., 2012; Sanzani et al., 2009; Tripathi and Dubey, 2004; Zhang et al., 2018; Zheng et al., 2021). Some earlier studies documented that *p*-hydroxybenzoic acid (pHBA) and its derivatives, which belong to plant-based phenolic compounds, significantly affected the growth of mycotoxigenic fungi (Huo et al., 2023; Laborda et al., 2019; Neto et al., 2015). The growth of *Aspergillus flavus* (*A. flavus*) was inhibited by pHBA, whereas that of *Alternaria alternata* (*A. alternata*) was inhibited by *p*-aminobenzoic acid (pABA) (Huo et al., 2023; Laborda et al., 2019). Another pHBA derivative, salicylic acid (o-hydroxybenzoic acid), also showed an antifungal activity against *Penicillium expansum* (*P. expansum*) (Neto et al., 2015). Additionally, gallic acid, another phenolic compound with antioxidant properties, is found in various plants including oak (Haslam and Cai, 1994). A previous study documented that the growth of *Fusarium solani* (*F. solani*) was substantially reduced by gallic acid (Nguyen et al., 2013).

Citral, a component of plant essential oils, is known to have a broad spectrum of antifungal inhibitory activities against a variety of plant pathogens such as *P. expansum* and *Penicillium digitatum* (*P. digitatum*) (OuYang et al., 2016; Tao et al., 2014; Wang et al., 2018). One previous study reported the combined inhibitory activity of citral and cinnamaldehyde on the growth of *P. expansum*, which was isolated from kiwifruits, and the expression of patulin biosynthetic genes (Wang et al., 2018). Also, it was much more effective than other plant-based natural compounds such as geraniol, eugenol, and limonene to the growth of *Alternaria* spp. and *Aspergillus* spp. (Kishore et al., 2007). Furthermore, citral was able to influence the expression of several mycotoxin biosynthetic genes in plant pathogenic fungi (Liang et al., 2015; Wang et al., 2019). However, the effects of these plant-based natural compounds on patulin synthesis by *P. paneum* and its growth remain largely unexplored. Thus, in the current study, we evaluated the effects of propyl paraben (n-propyl ester of pHBA), citral, and gallic acid on patulin accumulation by *P. paneum* OM1, which was isolated from a pear (cultivar Shingo) (Zhao et al., 2024) and its growth, and expression of major patulin biosynthetic genes in the fungal strain on YES (patulin supportive media). Moreover, we investigated the effect of propyl paraben on patulin accumulation by the fungal strain and its growth on pears.

## 2 Materials and methods

### 2.1 Chemicals and reagents

Patulin standard (≥98.0% purity), propyl 4-hydroxybenzoate (Propyl paraben, ≥99.0%), citral (95.0%), gallic acid (≥98.0%), Tween 80, and acetic acid were purchased from Sigma-Aldrich Co., (St. Louis, MO, USA). Ethyl acetate, glycerol (99.0%), and sucrose were obtained from Samchun chemical Co., (Seoul, South Korea). Ethyl alcohol (99.9%) and magnesium sulfate (heptahydrate [MgSO_4_⋅7H_2_O], ≥99.0%) were purchased from Duksan pure chemical Co., (Gyeonggi, South Korea). Anhydrous sodium sulfate (Na_2_SO_4_) and sodium carbonate (Na_2_CO_3_) were obtained from Samchun chemical Co., (Seoul, South Korea), and acetonitrile (ACN, ≥99.9%) were from J. T. Baker (Avantor Inc., Radnor, PA, USA).

### 2.2 Fungal strain and culture conditions

*P. paneum* OM1, a patulin-producing strain isolated from pear as described previously (Zhao et al., 2024), was used for this study. For fungal culture on agar plates containing different levels of 3 natural compounds, 10 μL of 2 × 10^7^ spores/mL were center-inoculated onto each YES (2% yeast extract, 15% sucrose, 0.1% MgSO_4_⋅7H_2_O) agar plate containing propyl paraben, citral, or gallic acid (1% of a final volume) at 3 different concentrations for each compound (10, 25, and 50 μg/mL dissolved in ethanol for propyl paraben, 100, 200, and 400 μg/mL diluted with ethanol for citral, 100, 250, and 500 μg/mL dissolved in ethanol for gallic acid), which was overlaid with a sterile cellophane membrane (GelAir Cellophane Support; Bio-rad, Hercules, CA, USA), and were incubated at 25 °C for 7 days. The addition levels of the three compounds into the media were chosen based on earlier studies regarding the fumonisin production from *Fusarium* spp., the growth of *Alternaria* sp., and the aflatoxin production from *Aspergillus* sp. (El-Nagar et al., 2020; Etcheverry et al., 2002; Liang et al., 2015). Fungal culture on YES agar plates containing only ethanol without any compound was used as a control.

For relative expression analysis of patulin biosynthetic genes on YES media containing propyl paraben, citral, or gallic acid, 10 μL of 2 × 10^7^ spores/mL were center-inoculated onto each YES agar plate containing propyl paraben, citral, or gallic acid (10 μg/mL for propyl paraben, 200 μg/mL for citral, 100 μg/mL for gallic acid), which was overlaid with a sterile cellophane membrane (GelAir Cellophane Support; Bio-rad), and were incubated at 25 °C for 6 days.

For fungal culture on pears, after the surface of pears had been disinfected with 70% ethanol, marked with a cross, and pierced with a small hole (5 mm) at the center using a sterile scalpel, 10 μL of spores (10^6^/mL) were inoculated into the hole on each half of a pear [cultivar Shingo, marketed primarily [85%] in South Korea and contains higher sugar, potassium, and water contents than Western pears (Lee et al., 2012)]. Then, 10 μL of propyl paraben (10 or 25 μg/mL) was applied into the hole with a cross, and the pears were incubated at 25 °C for 6 days. Ten μL of ethanol was used as a control. A half of a different pear was used for the inoculation of spores and application of propyl paraben (three halves in each treatment group). One half from nine halves of pear was randomly selected and assigned to the control or two treatment groups.

### 2.3 Measurement of fungal growth

Fungal growth was evaluated based on the diameter of colonies and dry weight of mycelia on YES agar plates or lesion diameter on pears. Radial mycelial growth was determined by assessing the diameter of each colony on YES plate or that of each colony on a pear lesion three times (horizontally, vertically, and diagonally). The dry weight of mycelia was assessed after its complete drying until a constant weight at 80 °C.

### 2.4 Patulin standard solutions and patulin extraction

After patulin powder was dissolved in ethyl acetate and the solutions were dried, 5 levels of patulin standards (0.1, 0.2, 0.5, 1.0, and 2.0 μg/mL) were prepared by resuspending each dried residue in the same volume of acidified distilled water (DW, pH 4.0) as that of ethyl acetate as described previously (Yu et al., 2025).

Patulin was extracted from entire mycelia plus agar in solid culture or a half piece of pears by the AOAC official method 995.10 with slight modifications (AOAC International, 2000) as published previously (Yu et al., 2025). Briefly, patulin in fungal cultures or pear was extracted twice with ethyl acetate and washed with 1.5% Na_2_CO_3_ solution. Then, it was dried over anhydrous Na_2_SO_4_ and completely evaporated under a gentle stream of N_2_ at 60 °C. After the dried residue was resuspended in acidified DW (pH 4.0), it was filtered through a sterile syringe filter with 0.22 μm polyvinylidene fluoride membrane (Hyundai Micro Co., Seoul, South Korea) for high-performance liquid chromatography (HPLC) analysis.

### 2.5 Assessment of accuracy and sensitivity of the analytical method for determination of patulin levels

The accuracy of the analytical method was assessed by the recovery rates of patulin extracted from samples, which were spiked with known concentrations (10 and 20 μg/mL) of patulin standard solutions to give 0.2, 0.5, and 1.0 μg/g pear of patulin levels, after the surface of pears had been sterilized with 2% sodium hypochlorite (NaOCl) and washed with DW. Extraction of patulin from the spiked samples was performed as described above. The recovery was calculated as follows:


Recovery=

Patulin⁢concentration⁢extracted⁢from⁢the⁢spiked⁢sample⁢x⁢ 100Patuin⁢concentration⁢used⁢for⁢spiking⁢the⁢sample

A limit of quantification (LOQ) and limit of detection (LOD) were used to evaluate the sensitivity of the analytical method for patulin quantification. LOQ and LOD were calculated using the slope (S) of the calibration curve and the standard deviation (SD) of the response by the following equations:


$$\text{LOQ}=\frac{10\,x\,\mathrm{SD}}{\text{S}}$$

$$\text{LOD}=\frac{3.3\,x\,\mathrm{SD}}{\text{S}}$$

The LOQ and LOD for patulin were 0.071 and 0.023 μg/mL, respectively.

### 2.6 HPLC-UVD analysis

An HPLC system (LC-20AD, Shimadzu; Tokyo, Japan) coupled with UV detector (UVD, SPD-20A, Shimadzu; Tokyo, Japan) was used to detect and quantify patulin at 276 nm as described previously (Zhao et al., 2024). The mobile phase (10% ACN [ACN: DW = 10:90, v/v]) was delivered onto a ZORBAX Eclips plus C18 column (5 μm particle size, 4.6 mm × 250 mm, Agilent; Santa Clara, CA, USA), which was maintained at 30 °C, at a flow rate of 0.5 mL/min to separate analytes. The injection volume of the samples was 20 μL.

The calibration curve of patulin was constructed by plotting five patulin concentrations (*x* axis) versus peak areas (*y* axis) after HPLC analysis of the five levels of patulin standards (0.1, 0.2, 0.5, 1, and 2 μg/mL). The linearity of the standard curve was evaluated by linear regression analysis and determined by a coefficient of determination (r^2^). The curve had 0.999 of r^2^ value (Supplementary Figure 1).

### 2.7 Relative gene expression analysis

After culture on YES agar plates for 3 or 6 days as described above, the mycelia were immediately frozen under liquid N_2_. Total RNA was extracted from the mycelia using RNeasy Mini Kit (Qiagen, Hilden, Germany) according to the manufacturer’s protocol after the frozen mycelia were ground with a mortar and pestle into a fine powder as described previously (Zhao et al., 2024). The RNA quality was examined by an Agilent 2100 Bioanalyzer (Agilent, Santa Clara, CA, USA). Primer pairs specific to five patulin biosynthetic genes (*patK*, *patL*, *patN*, *patG*, and *patE*) were designed using Primer3Plus based on the genome sequence of *P. paneum* OM1 (Table 1).

**TABLE 1:** Primer sequences used for RT-qPCR analysis.

Gene	Orientation	Primer sequence (5′→ 3′)	Amplicon size (bp)
*patK*	Forward Reverse	TTGCCGGTCCAGTATTCCAC CCAACTCTGAACGGCCTCAT	146
*patL*	Forward Reverse	CTCCACTTTCTCCGCCAACT GCTGCTTGTCATCGCATCTG	149
*patN*	Forward Reverse	ACTGGACGAGGGCATCTTTG AATGTATCCTACTGCGCCCG	133
*patG*	Forward Reverse	CATCTCGTCAAACCCGGTCA CGAGGAAGTCTGTGAAGCGT	125
*patE*	Forward Reverse	GGATCTTTTCCCACGCCAGA CACGCAGTCAGCCCTTCTAA	119
*β-tubulin*	Forward Reverse	GGTAACCAAATCGGTGCTGCTTTC ACCCTCAGTGTAGTGACCCTTGGC	291

The cDNA synthesis was carried out using PrimerScript RT reagent Kit with gDNA Eraser (TakaRa Bio Inc., Shiga, Japan), and RT-qPCR amplification was conducted using 2× Real-Time PCR Master Mix (BIOFACT, Daejeon, South Korea) following a procedure provided by the manufacturer as stated previously (Zhao et al., 2024). The RT-qPCR reaction was performed using CFX96TM Touch Real-Time PCR Detection System (Bio-Rad, Hercules, CA, USA) as follows: 95 °C for 2 min, followed by 40 cycles of 95 °C for 10 s, 60 °C for 15 s, and 72 °C for 10 s. After the final amplification cycle, a melting curve analysis of the amplicons was performed by a 0.5 °C increase per s from 60 °C to 95 °C to verify the formation of a single amplicon. The RT-qPCR data were analyzed using CFX Maestro software (Bio-Rad, Hercules, CA, USA), and relative gene expression levels were calculated by the 2^–ΔΔCT^ method together with β-tubulin gene (GenBank accession number AF003248.1) for normalization (Livak and Schmittgen, 2001). The RT-qPCR analysis was carried out with three independent biological samples and two replicates were analyzed for each biological sample.

### 2.8 Statistical analyses

Statistical analyses were performed by a one-way or two-way analysis of variance (ANOVA), which was followed by Duncan’s test as a *post hoc* analysis. Data were represented as the mean ± standard deviation (SD) using SPSS 26.0 (SPSS Inc., Chicago, IL, USA). A *p*-value < 0.05 was considered statistically different.

## 3 Results

### 3.1 Effects of propyl paraben, citral, and gallic acid on patulin production by *P. paneum* OM1 and its growth

To investigate the inhibitory effects of propyl paraben, citral, gallic acid on the levels of patulin produced by *P. paneum* OM1, we cultured the fungal strain on YES agar plates containing 3 levels of 3 different natural compounds (propyl paraben, citral, or gallic acid) for 7 days. In all 3 treatment groups, the amounts of patulin produced by the fungal species remarkably decreased relative to the control group (99.9% for propyl paraben, 100% for citral, 37.8% for gallic acid) after adding the maximum level of each compound (50 μg/mL of propyl paraben, 400 μg/mL of citral, 500 μg/mL of gallic acid) (Figure 1). In particular, patulin was not detected due to suppression by 25 μg/mL of propyl paraben. However, of the 3 treatment groups, treatment with propyl paraben or citral continuously reduced patulin production with increased concentrations of compounds in media compared to the control group, whereas gallic acid treatment led to similar level of patulin production in media with 3 levels of compound (32.9%–37.8% reduction) (Figure 1).

**FIGURE 1:**
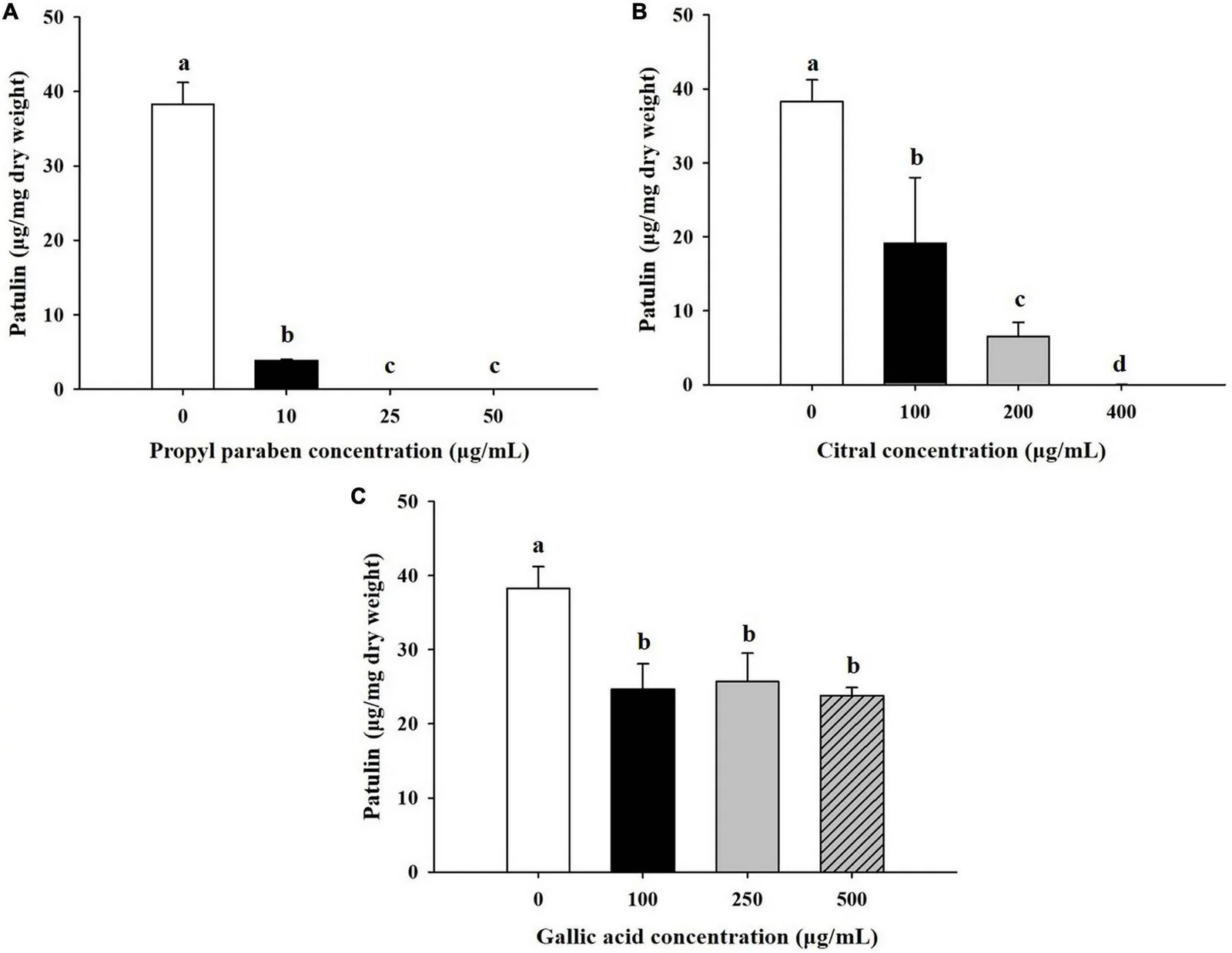
Effects of 3 natural compounds on patulin accumulation by *P. paneum* OM1 on YES agar plates after 7 days of culture at 25 °C. **(A)** Propyl paraben (10, 25, and 50 μg/mL), **(B)** citral (100, 200, and 400 μg/mL), and **(C)** gallic acid (100, 250, and 500 μg/mL). The levels of patulin were measured in triplicate (three plates from each group and three replicates from each plate). Data are represented as the mean ± standard deviation. Different letters indicate statistically significant differences (*P* < 0.05).

Also, we analyzed the effects of the 3 compounds on the growth rates of the strain. Similar to the pattern of patulin production described above, propyl paraben or citral treatment decreased the fungal growth rates relative to the control as the compound concentration increased although fungal growth in treatment with the lowest level of compound was not statistically different from that of the control (*p* < 0.05) (Figures 2A, B). The growth rates, which were based on the diameter of fungal colonies, were substantially reduced (33.1% for propyl paraben, 42.8% for citral) compared to the control group after addition of the maximum amounts for the 2 compounds (50 μg/mL of propyl paraben, 400 μg/mL of citral). However, a slightly different result was observed in gallic acid treatment group, in which the growth rates of the fungal species were not affected by any level of the compound (Figure 2C). Also, the growth rates, which were based on the dry weight of fungal mycelia, showed a similar pattern to those from colony diameter. The growth rates were progressively reduced relative to the control group with the increasing levels of compounds except for gallic acid treatment group (Supplementary Figure 2). The addition of the maximum levels of the 2 compounds considerably inhibited the mycelial dry weights (36.7% for propyl paraben, 54.3% for citral), which are similar to the data based on colony diameter described above (Supplementary Figures 2A, B). Likewise, the growth rates in gallic acid treatment group, based on the fungal dry weight, did not exhibit any statistically significant difference from the control group (*p* < 0.05) although they were all slightly reduced (Supplementary Figure 2C). On the whole, our data demonstrated dose-dependent decreases in patulin levels and inhibition of fungal growth by propyl paraben and citral relative to the control group. Since the patulin production was markedly reduced by propyl paraben, citral, or gallic acid at the concentrations of 10, 200, or 100 μg/mL, respectively, we selected these levels for subsequent RT-qPCR analyses.

**FIGURE 2:**
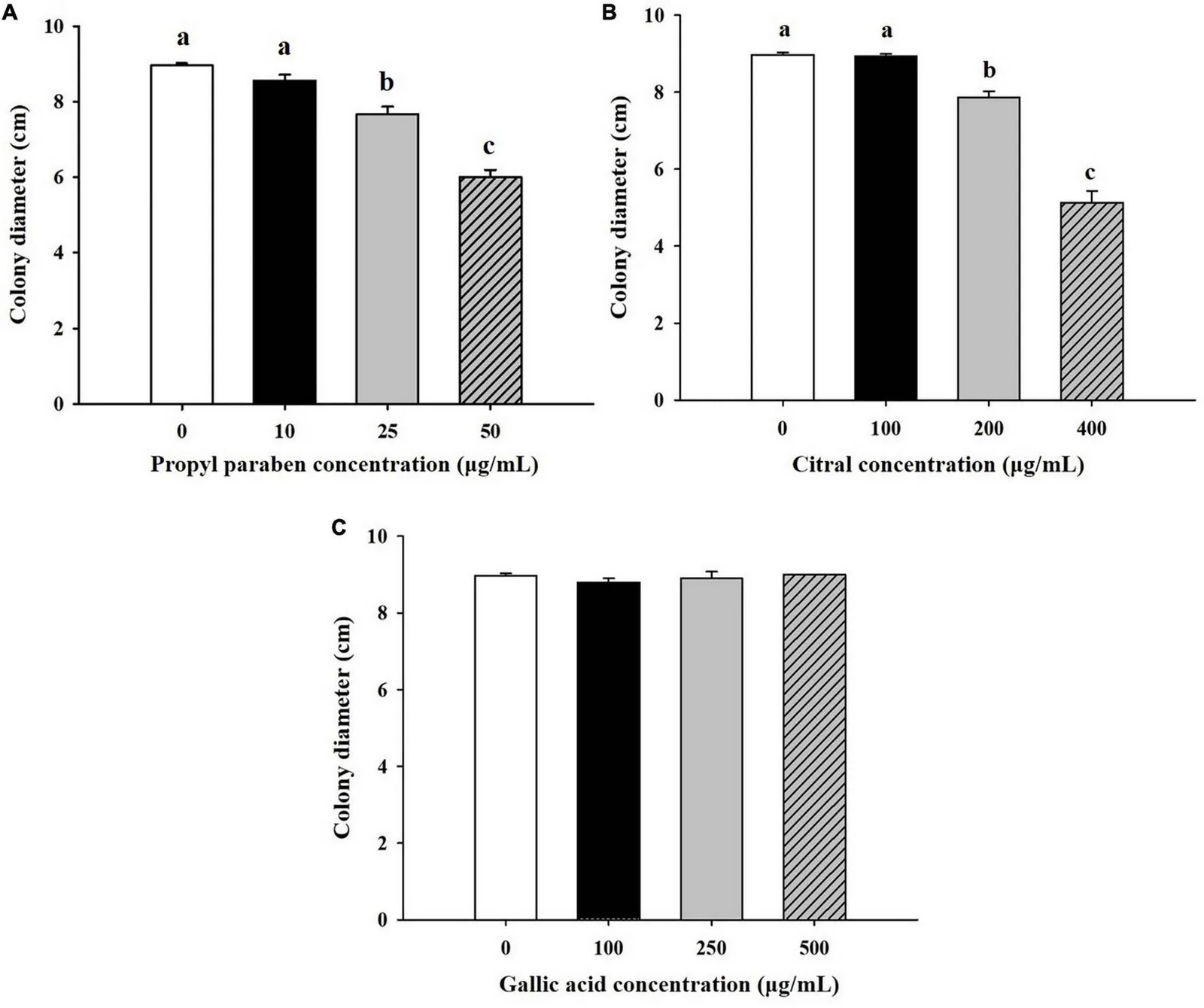
Effects of 3 natural compounds on colony diameter of *P. paneum* OM1 on YES agar plates after 7 days of culture at 25 °C. **(A)** Propyl paraben (10, 25, and 50 μg/mL), **(B)** citral (100, 200, and 400 μg/mL), and **(C)** gallic acid (100, 250, and 500 μg/mL). The colony diameter of mycelia was analyzed in triplicate (three plates from each group and three replicates from each plate). Data are represented as the mean ± standard deviation. Different letters denote statistically significant differences (*P* < 0.05).

### 3.2 Effects of propyl paraben, citral, and gallic acid on relative expression levels of patulin biosynthetic genes in *P. paneum* OM1

Because the decreases in patulin production levels by the 3 compounds may have been due to reduced expression levels of patulin biosynthetic genes in *P. paneum* OM1, we analyzed the relative expression levels of 5 key patulin biosynthetic genes (*patK*, *patL*, *patN*, *patG*, and *patE*) in the fungal strain on YES media containing each compound. In all 3 treatment groups, the 5 gene expression levels decreased relative to the control group after 3 days of incubation (Figure 3). Although no statistical differences were observed among 3 treatment groups, the propyl paraben treatment group tended to show slightly lower expression levels of the 5 genes than the other 2 treatment groups on the 3rd day. As expected, the gene expression levels in the control group increased remarkably on the 6th day compared to those on the 3rd day, whereas the levels in all 3 treatment groups were suppressed on the 6th day compared to those in the control group (Figure 3). However, the expression level of *patE* in the gallic acid treatment group was comparable to that in the control group on the 6th day.

**FIGURE 3:**
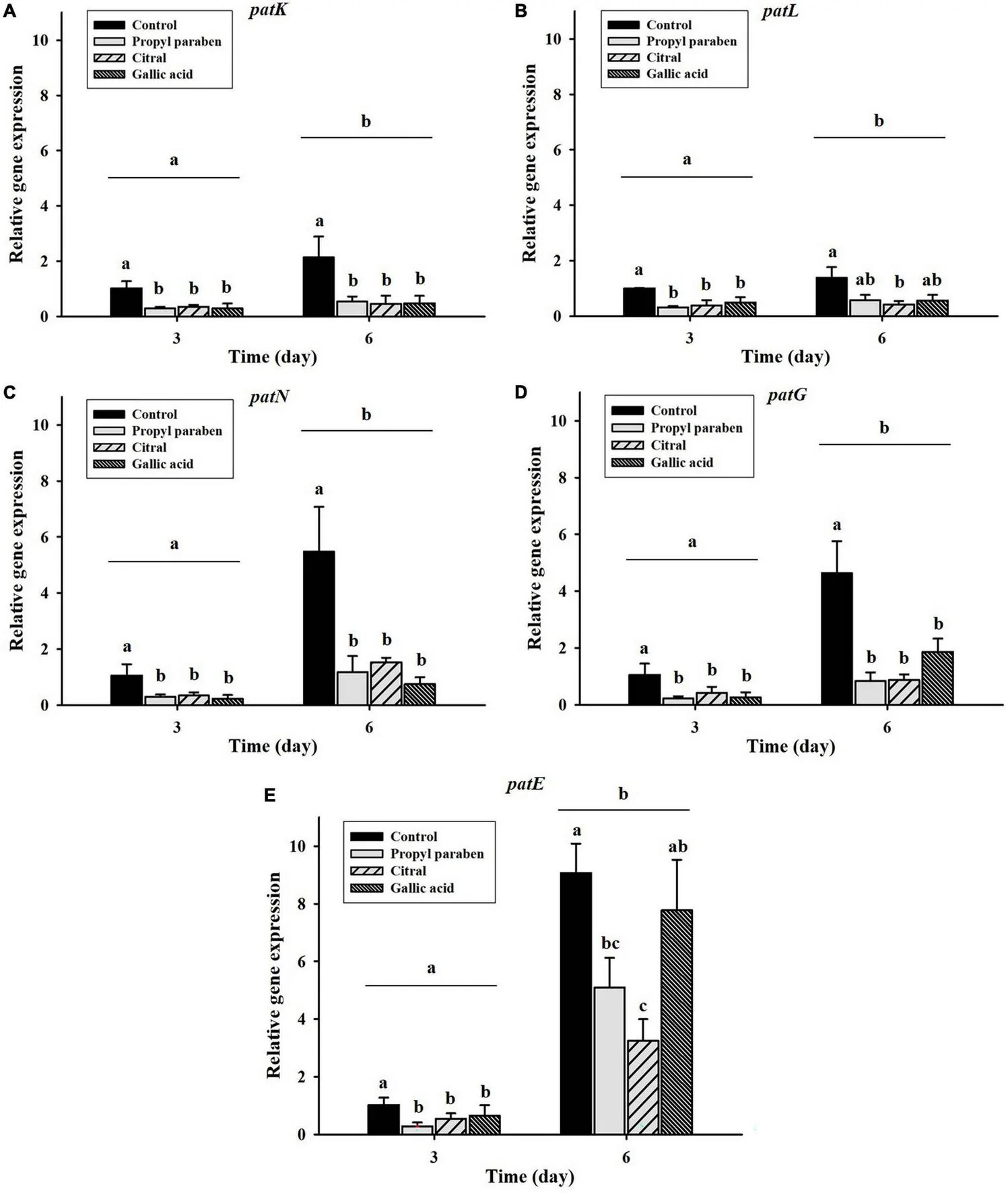
Effects of propyl paraben (10 μg/mL), citral (200 μg/mL), and gallic acid (100 μg/mL) on the expression of 5 major patulin biosynthetic genes in *P. paneum* OM1 on YES agar plates after 3 and 6 days at 25 °C. **(A)** *patK*, **(B)** *patL*, **(C)** *patN*, **(D)** *patG*, and **(E)** *patE*. The expression levels of 5 genes were analyzed in triplicate (three plates from each group and two replicates from each plate). The expression levels were normalized by that of β-tubulin gene. Data are represented as the mean ± standard deviation. Different letters on the same day indicate statistically significant differences (*P* < 0.05). There is no statistical difference in the treatment × time interaction in panels **(A,B)**, whereas there is a statistical difference in the interaction between the two factors in panels **(C–E)**.

Also, the growth inhibition by the selected concentration of compounds was similar to those described above as shown in Figures 4A, B. As expected, the levels of patulin in all 3 treatment groups were extremely low on the 3rd day, but increased sharply until the 6th day (Figure 4C and Supplementary Figures 3A–E). This is consistent with the elevated expression levels of the 5 patulin biosynthetic genes on the 6th day described in Figure 3. In particular, in the propyl paraben treatment, the largest decrease in the patulin production level was observed among the 3 treatments relative to the control after 6 days of incubation (approximately 73% reduction) (Figure 4C). Also, the patulin level was 42% lower in the citral treatment than in the control, whereas the level was slightly (10%) lower in the gallic acid treatment than in the control. Since propyl paraben inhibited the expression of patulin biosynthetic genes and patulin production in *P. paneum* OM1 and its growth even at a low concentration, we chose propyl paraben for further experiments.

**FIGURE 4:**
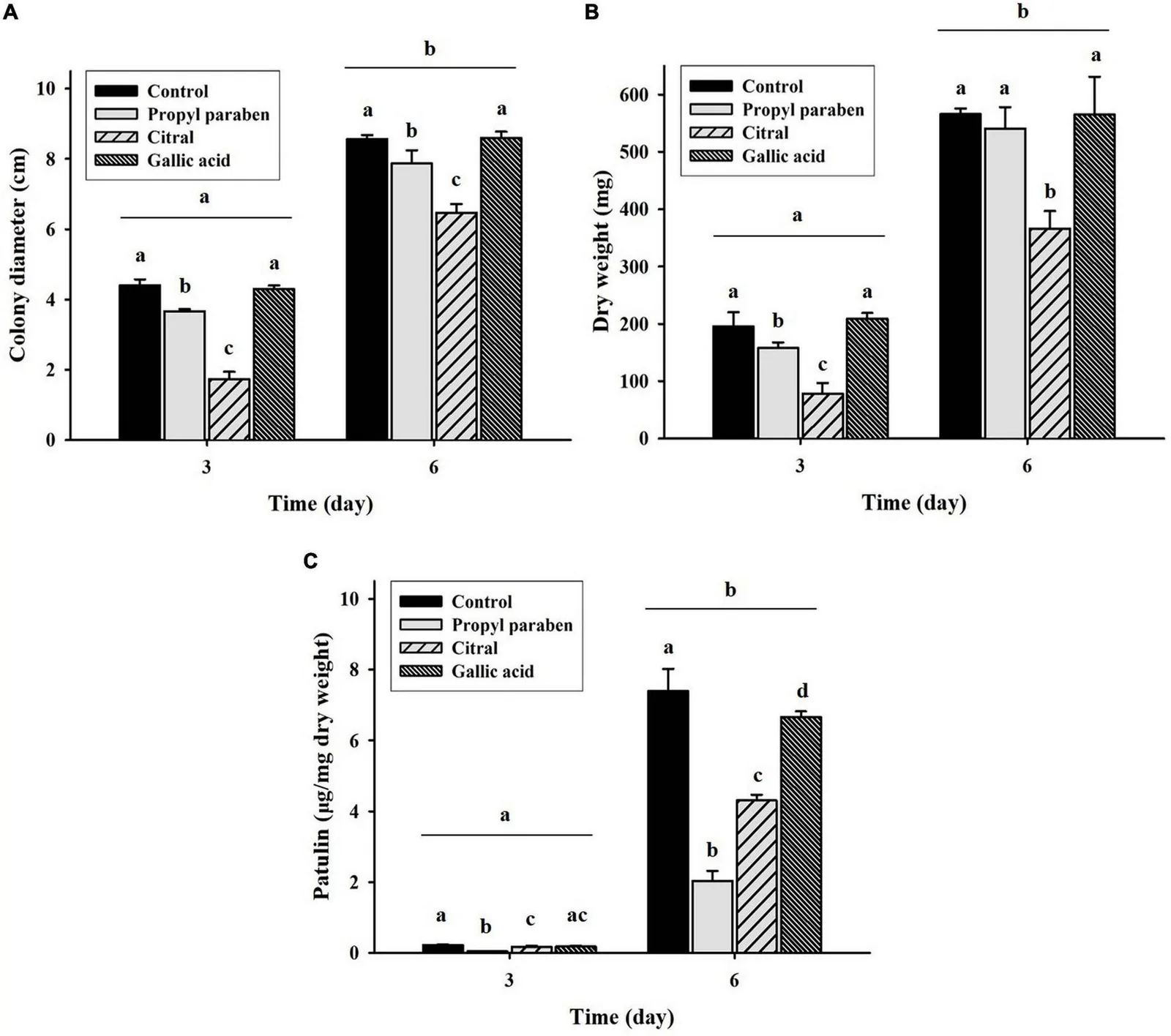
Effects of propyl paraben (10 μg/mL), citral (200 μg/mL), and gallic acid (100 μg/mL) on the growth of *P. paneum* OM1 and its patulin accumulation on YES plates over 6 days at 25 °C. **(A)** Colony diameter, **(B)** dry weight, and **(C)** patulin production. The colony diameter and dry weight of mycelia and levels of patulin were measured in triplicate (three plates from each group and three replicates from each plate). Data are represented as the mean ± standard deviation. Different letters on the same day indicate statistically significant differences (*P* < 0.05). There is no statistical difference in the treatment × time interaction in panels **(A,B)**, whereas there is a statistical difference in the interaction between the two factors in panel **(C)**.

### 3.3 Effects of propyl paraben on patulin production by *P. paneum* OM1 and its growth on a pear

Since *P. paneum* OM1 was previously isolated from pear as described above, we assessed the effects of propyl paraben on patulin accumulation by the strain and its growth on pears (cultivar Shingo). We observed the lesion area treated with higher amount of propyl paraben was reduced by 57% relative to that in the control (1.05 and 0.7 cm in the control and propyl paraben treatment groups, respectively, for average diameter, 0.88 and 0.38 cm^2^ in the control and propyl paraben treatment groups, respectively, for average lesion area) (Figure 5A). Also, amounts of patulin produced by the fungal strain decreased on the 6th day after 2 dosages of propyl paraben (0.1 or 0.25 μg/half of a pear) were applied on pears. In particular, in the application of 0.25 μg propyl paraben onto half of a pear, patulin produced by the strain in pears was below the detection limit, whereas the application of 0.1 μg of propyl paraben/half of a pear resulted in 70% decrease in its patulin production compared to the control (LOD for patulin: 0.038 μg/mL, LOQ for patulin: 0.116 μg/mL) (Figure 5B and Supplementary Figures 3F, G).

**FIGURE 5:**
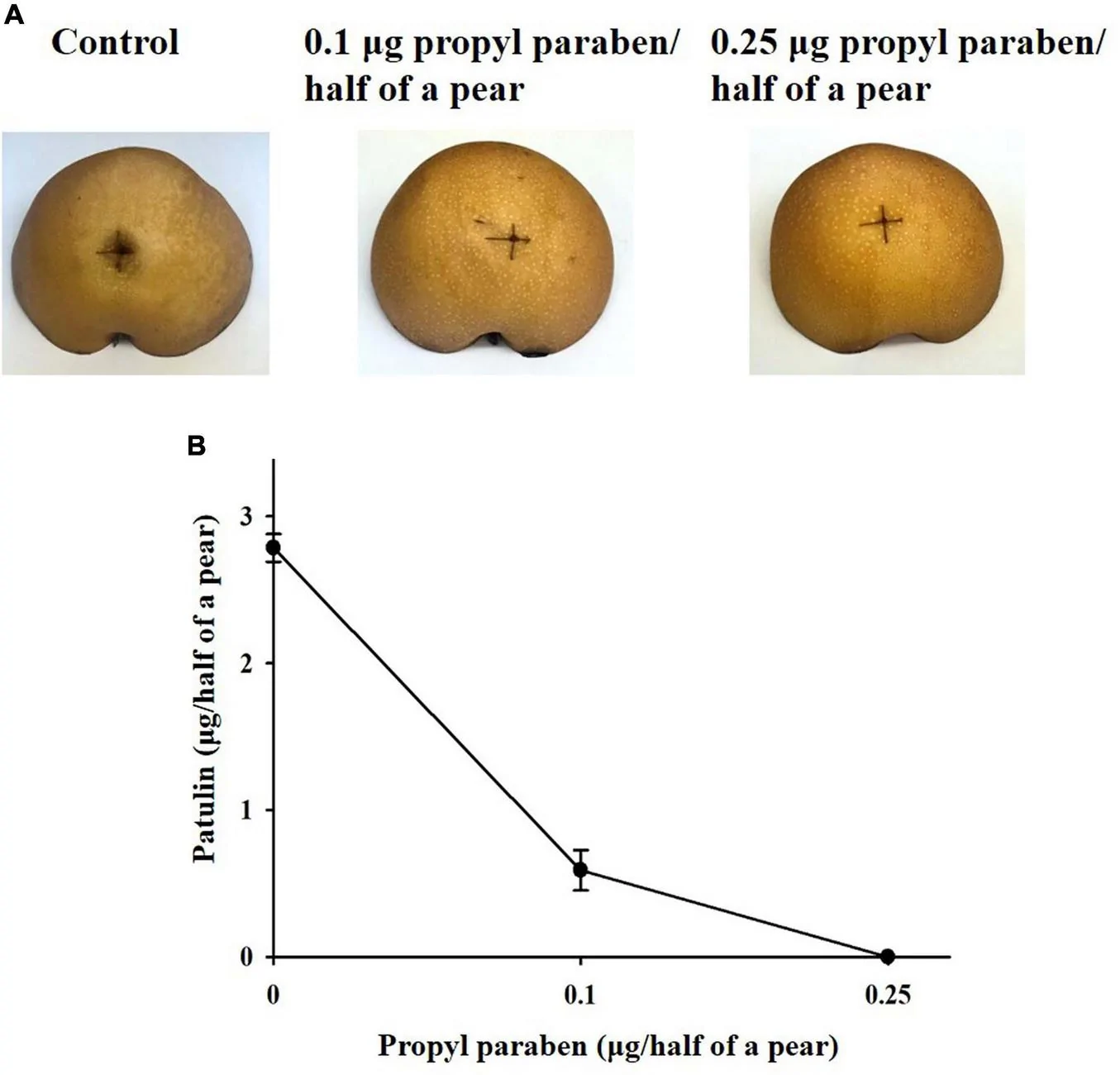
Effects of 2 different levels of propyl paraben on the growth of *P. paneum* OM1 and its patulin accumulation on pears after 6 days at 25 °C. **(A)** Supplementation of 0, 0.1, and 0.25 μg propyl paraben into the hole with a cross on half of a pear, which was artificially infected with the fungal strain, and **(B)** its patulin production levels. One half from nine halves of pear (a half from each pear, three halves in each group) was randomly selected and assigned to the control or two treatment groups. The levels of patulin were analyzed in triplicate (three halves of pear from each group and three replicates from one sample). Data are represented as the mean ± standard deviation.

On the other hand, the recovery for patulin in pears was in the range of 75.38%–81.12% (Table 2 and Supplementary Figures 3H, I). These recovery values were higher than 75%, which is consistent with the recovery (75%–105%) approved by Official Journal of the European Union (European Commission (EC), 2006). Thus, this result indicates that the analytical method yielded good recoveries of patulin from pear.

**TABLE 2:** Recovery of patulin extracted from pears spiked with patulin standard solutions (10 and 20 μg/mL).

Sample	Spiking level (μ g/mL)	Recovery (%)
Pear	0.2	81.12 ± 3.37
	0.5	78.70 ± 5.53
	1.0	75.38 ± 3.87

Recoveries were measured in triplicate (three halves of pear from each group and three replicates from one sample). One half from nine halves of pear (a half from each pear, three halves in each group) was randomly selected and assigned to each group. Data are represented as the mean ± standard deviation.

## 4 Discussion

Patulin is a mycotoxin that causes neurotoxic, genotoxic, and carcinogenic effects to humans and animals after consumption of agricultural products contaminated with the toxin. To date, available data are scarce regarding the influence of natural compounds on growth of patulin-producing fungi, patulin production, and patulin biosynthetic gene expression. Also, in recent years, the use of synthetic fungicides has been restricted due to public concerns to the toxicities of their residues on food and the emergence of fungicide-resistant fungal strains (Islam et al., 2024; Qin et al., 2021). Thus, in the current study, we selected three natural compounds (propyl paraben, citral, and gallic acid), which have antimicrobial and antioxidant activities and belong among substances affirmed as generally recognized as safe (GRAS) by US Food and Drug Administration (FDA), for its practical application. This study aimed to investigate the effects of propyl paraben, citral, and gallic acid on patulin production and expression levels of patulin biosynthetic genes in *P. paneum* OM1 and its growth on YES. Our results showed propyl paraben and citral significantly affected both patulin production by the fungal strain and its growth in a dose-dependent manner, whereas gallic acid affected only its patulin production. These results are consistent with those from previous studies although they focused on the growth of different mycotoxigenic fungi and their mycotoxin production (Dix, 1979; Etcheverry et al., 2002; Hughes et al., 2007; Huo et al., 2023; Laborda et al., 2019; Liang et al., 2015; Wu et al., 2009). Hughes and co-workers’ study documented that pHBA, a de-esterified derivative of propyl paraben, inhibited the mycelial growth of *Penicillium commune* by 10% on minimal media containing glucose and pHBA (690 μg/mL) (Hughes et al., 2007). Another study showed that the dry weight of a *Fusarium oxysporum* strain was inhibited by 16% on potato dextrose agar (PDA) containing 200 μg/mL of pHBA (Wu et al., 2009). Also, Huo and collaborators described that 140 μg/mL of pHBA suppressed the colony diameter of *A. flavus* by approximately 70% (Huo et al., 2023). It indicates that pHBA is an antifungal compound against a broad spectrum of mycotoxigenic fungi. Also, pABA, a pHBA derivative, showed a similar result to those of pHBA on fungal growth. Laborda and collaborators reported that colony diameter of *A. alternata* was reduced by 31% on PDA containing 137 μg/mL of pABA (Laborda et al., 2019). In addition, 180 μg/mL of propyl paraben completely inhibited fumonisim production by *Fusarium verticillioides* or *Fusarium proliferatum* (Etcheverry et al., 2002). In their study, the growth of *Fusarium* spp. was totally suppressed by 20-fold higher level of propyl paraben than that in our study. It may have come from strain dependent sensitivity to the compound.

On the other hand, several previous studies reported the antifungal and anti-mycotoxigenic effects of citral (Liang et al., 2015; Wang et al., 2019). One earlier study showed that growth of *Penicillium italicum* (*P. italicum*) was inhibited by 65% or 100% on PDA plates containing 210 or 422 μg/mL of citral, respectively (Tao et al., 2014), and another study described that mycelial growth of *P. expansum* was reduced by 68% in modified Czapek-Dox media containing 150 μg/mL of citral (Venturini et al., 2002), which are in agreement with our results. In addition, Liang and colleagues’ study reported that both aflatoxin B1 production by *A. flavus* and its growth were completely inhibited in YES containing approximately 420 μg/mL of citral, whereas only the aflatoxin B1 production decreased by 42% in the same media containing 85 μg/mL of the compound compared to the control (Liang et al., 2015), which is similar to our results although it is a different mycotoxin from aflatoxin B1. In our study, when 400 μg/mL of citral was added to YES, patulin accumulated by *P. paneum* OM1 was not detected and the addition inhibited its growth partially (42.8% decrease in colony diameter, 54.3% decrease in dry weight), while the addition of 100 μg/mL of citral to YES inhibited patulin production by 50% but resulted in no growth inhibition, based on both colony diameter and dry weight. Also, in a previous study, the authors described that citral induced the disturbance of cell integrity via inhibition of ergosterol biosynthesis and fatty acid metabolism (Wang et al., 2019), which are essential for fungal cell membrane integrity (Dwivedy et al., 2016; OuYang et al., 2016; Wang et al., 2019). Based on the previous study, it may be hypothesized that citral reduced patulin production by inhibiting the fungal growth partially through altered cell membrane in our study. Additionally, the Wang and co-workers’ study documented that the growth of *A. alternata* was completely inhibited in potato dextrose broth (PDB) containing approximately 200 μg/mL of citral, and more than 97% of alternariol production was suppressed in the same media containing 112 μg/mL of citral (Wang et al., 2019). In our study, the growth of *P. paneum* OM1 on YES supplemented with 200 μg/mL of citral was inhibited by 12% and 20% on the basis of colony diameter and mycelial dry weight, respectively, whereas 50% of patulin production decreased by 100 μg/mL of citral, which are slightly different from their study. The discrepancy might have been due to higher sensitivity of the cell membrane of *A. alternata* on PDB to citral than that of *P. paneum* OM1 on YES through alteration of ergosterol biosynthesis, which is one of the possible mechanisms for the altered cell membrane synthesis. Interestingly, in our study, the data revealed that citral inhibited the growth of *P. paneum* OM1 more significantly than propyl paraben relative to the control after 6 days of incubation, whereas propyl paraben suppressed its patulin production more effectively than citral. Huo and colleagues documented that pHBA inhibited the growth of *A. flavus* by blocking its cytokinesis (Huo et al., 2023). We assume in our study that the inhibitory effect of propyl paraben on growth of *P. paneum* OM1 via its altered cytokinesis, a possible mechanism of propyl paraben, was slightly weaker than that of citral on the fungal growth through disturbed ergosterol biosynthesis, based on the similar levels of down-regulated expression of patulin biosynthetic genes.

Also, our data showed that there was no decrease in the growth of *P. paneum* OM1 on YES containing 100, 250, or 500 μg/mL of gallic acid, which is consistent with one earlier study (Dix, 1979). One study reported that 5 different *Penicillium* species including a *P. expansum* strain showed only slight (0%–20%) reduction in their growth on Czapek-Dox agar plates containing 0.5% gallic acid or in minimal media containing the same level of gallic acid, unlike other fungal species such as *Aspergillus* sp., *Fusarium* sp., and *Alternaria* sp. (Dix, 1979). However, these results slightly differ from another study. Nguyen and co-workers’ study described that the growth of *F. solani* was reduced by 33% or 75% in a dose-dependent manner on agar plates containing 100 or 500 μg/mL of gallic acid (Nguyen et al., 2013). The reason for this may be the use of different fungal strain. In the Dix’s study, the author described that gallic acid is converted to incomplete oxidation products in *Penicillium* spp., which are tolerant to the products, because they have low polyphenol oxidase activity, whereas it is converted to toxic oxidation products in other fungal species because they have high polyphenol oxidase activity (Dix, 1979).

Besides, our data revealed that expression of the 5 key patulin biosynthetic genes (*patK*, *patL*, *patN*, *patG*, and *patE*) were down-regulated in *P. paneum* OM1 on YES media containing propyl paraben, citral, or gallic acid except for *patE* under the addition of gallic acid. These findings are also in accordance with those from earlier studies (Liang et al., 2015; Wang et al., 2019). In their studies, the expression of aflatoxin B1 biosynthetic and alternariol biosynthetic genes was down-regulated in *A. flavus* by 85 μg/mL of citral and *A. alternata* by 112 μg/mL of citral, respectively (Liang et al., 2015; Wang et al., 2019). Also, Huo and co-workers’ study reported that expression of major aflatoxin biosynthetic genes in *A. flavus* was down-regulated by 690 μg/mL of pHBA (Huo et al., 2023). In our study, when combined with decreased levels of patulin in *P. paneum* OM1 described above, these data suggest that the reduced patulin production resulted from the suppression of patulin biosynthetic gene expression at the transcriptional level in the strain. Moreover, in Wang and collaborators’ study, the authors also described that citral induced ROS imbalance and decreased intracellular total antioxidant capacity and catalase activity in *A. alternata*, leading to down-regulation of the expression of alternariol biosynthetic genes such as *pksI* and *omtI* as described above (Wang et al., 2019). In another study, Kim and colleagues hypothesized that ROS scavenging ability of phenolic compounds decreases oxidative stress and in turn reduces aflatoxin production in *A. flavus* (Kim et al., 2005). Thus, based on previous studies, it may be hypothesized that in our study citral or propyl paraben decreased intracellular ROS in *P. paneum* OM1, resulting in reduced patulin biosynthesis by suppressing the expression of patulin biosynthetic genes.

In particular, the expression of *patL*, which encodes C6 transcription factor, was down-regulated together with other patulin biosynthetic genes by addition of each natural compound into the media. It also suggests that the natural compounds inhibited patulin synthesis by down-regulating the expression levels of the four patulin biosynthetic genes via the suppression of the expression of *patL*. Surprisingly, the expression level of *patE* under the addition of gallic acid into the media was comparable to that of the control without any compound after 6 days of culture. One possible explanation for this is that the expression of the early (*patK* and *patG*) and middle (*patN*) genes in the patulin biosynthetic pathway are suppressed but the expression of the late gene (*patE*) is not suppressed directly by gallic acid when considering the patulin biosynthetic pathway (Li et al., 2019). Notably, patulin production by *P. paneum* OM1 decreased by only 37.8% even at the highest level of gallic acid (500 μg/mL) although its patulin production was almost completely suppressed at the highest level of propyl paraben (50 μg/mL) or citral (400 μg/mL). This might have been due to the unsuppressed expression level of *patE* (encodes oxidoreductase that catalyzes the last step in the patulin biosynthetic pathway).

In this study, although gallic acid did not affect the growth of *P. paneum* OM1 as a primary metabolism as described above, it decreased its patulin production by down-regulating the expression of the patulin biosynthetic genes. These results are in line with those from a previous study (Sanzani et al., 2009). Sanzani and co-workers reported that the growth of *P. expansum* was not influenced by 10 μg/mL of umbelliferone, a phenolic compound with antioxidant activities, but its patulin production was reduced by acting on the patulin biosynthetic genes (Sanzani et al., 2009). In addition, our study showed that gallic acid decreased patulin levels produced by the fungal strain in a dose-independent manner, which are slightly different from inhibition of its patulin production by propyl paraben or citral. This might have been attributed to no inhibition of expression of *patE* by gallic acid in contrast to stronger inhibition of expression of the same gene by propyl paraben or citral. Also, previously, it was reported that fungal growth was not affected in spite of reduction of secondary metabolite production (Demain, 1996). Our data strengthen the fact that gallic acid does not significantly affect the growth of *Penicillium* sp. and that reduction of secondary metabolite production has little effect on fungal growth.

Finally, we analyzed the influence of propyl paraben on its patulin production and growth on pears to evaluate the practical application of the compound because propyl paraben showed the strongest inhibitory effect on both patulin accumulation by *P. paneum* OM1 and its growth on patulin supportive media even at a low concentration among three natural compounds. Our data showed that propyl paraben (0.25 μg/half of a pear) inhibited both growth of *P. paneum* OM1 and its patulin accumulation on pears, resulting in 57% reduced pear lesions. One earlier study obtained a similar result to that from our study (Huo et al., 2023). Huo and co-workers documented that 690 μg/mL of pHBA spray reduced the lesion caused by *A. flavus* on kiwifruit by 46% when 10^4^ of spores were inoculated on each fruit and it was incubated at 26 °C for 5 days (Huo et al., 2023). However, despite the similar results in two studies, in their study, the authors sprayed a high level of pHBA, a derivative of propyl paraben, onto kiwifruits inoculated with *A. flavus*, whereas we applied a very low amount of propyl paraben onto pears inoculated with *P. paneum* OM1. It may suggest higher inhibitory effect of propyl paraben than pHBA on mycotoxigenic fungi, more sensitivity of *P. paneum* OM1 to propyl paraben than pHBA, or both. Furthermore, a previous study documented that increased levels of intracellular ROS down-regulate cAMP-PKA signaling pathway in *Aspergillus parasiticus*, resulting in increased expression of antioxidant genes including catalase gene and secondary metabolite biosynthetic genes including aflatoxin biosynthetic genes (Hong et al., 2013). Given that ROS scavenging ability of phenolic compounds reduces oxidative stress and in turn decreases aflatoxin production in *A. flavus* described above, it may be hypothesized that in our study propyl paraben with antioxidant activity reduced ROS in *P. paneum* OM1, leading to a decrease in patulin biosynthesis by down-regulating the expression of patulin biosynthetic genes. Additionally, propyl paraben may have partially decreased patulin production by inhibiting its growth through altered cytokinesis.

To date, several previous studies have reported on the control of the growth of *Penicillium* spp. and their patulin production by natural compounds, but these have been largely limited to *P. expansum*, *P. digitatum*, and *P. italicum* (Fan et al., 2014; OuYang et al., 2016; Sanzani et al., 2009; Tao et al., 2014; Zheng et al., 2015). To our knowledge, this is the first report on controlling patulin contamination of pears by a *P. paneum* strain with food-grade natural compounds.

In this study, we demonstrated that the strong inhibitory effects of propyl paraben and citral on patulin accumulation by *P. paneum* OM1 and its growth on patulin supportive media. Given their high antifungal activities and GRAS grade status, propyl paraben and citral could be promising natural compounds for practical applications in the fruit industry. Moreover, the combination of propyl paraben and citral could exert synergistic effects in prevention of patulin production by *P. paneum* OM1 and its growth on pome fruits. Future research will be focused on how these natural compounds suppressed the expression of patulin biosynthetic genes in *P. paneum* OM1.

## 5 Conclusion

In the present study, our data demonstrated that 2 compounds (propyl paraben and citral) out of 3 plant-based natural compounds inhibited the growth of *P. paneum* OM1 and that the compounds along with gallic acid down-regulated the expression of 5 key patulin biosynthetic genes (*patK*, *patL*, *patN*, *patG*, and *patE*) except for *patE* under gallic acid supplementation, which resulted in the reduction of patulin biosynthesis in the fungal strain. In particular, of the three GRAS grade compounds, propyl paraben and citral decreased patulin production in a dose-dependent manner (0–25 μg/mL for propyl paraben, 0–400 μg/mL for citral) and also inhibited its growth significantly at 25 and 400 μg/mL, respectively. Additionally, the application of 0.25 μg of propyl paraben onto a half of a pear, which was artificially inoculated with *P. paneum* OM1, confirmed the complete inhibition of its patulin production and 57% inhibition of its growth on them. These findings could be helpful in development of effective natural compounds to alleviate and control patulin accumulation by *P. paneum* OM1 on pears during postharvest storage.

## Data Availability

The raw data supporting the conclusions of this article will be made available by the authors, without undue reservation.
