# Supplementary material for: Inhibitory effects of propyl paraben, citral, and gallic acid on the growth, patulin biosynthesis, and patulin biosynthetic gene expression of Penicillium paneum OM1
Source: Front Microbiol. 2026 Sep 28;17:1949526. doi: 10.3389/fmicb.2026.1949526 (PMC13654974; doi:10.3389/fmicb.2026.1949526)
Supplement: Supplementary file 1 [file Supplementary_file_1.docx]

Supplementary Material

# 1 Supplementary Figures

**Supplementary Figure 1**

Calibration curve of patulin standards for quantification of patulin by HPLC-UVD. Five levels of patulin standard solutions were prepared in the range of 0.1, 0.2, 0.5, 1.0, and 2.0 μg/mL. Each solution was injected into HPLC-UVD in triplicate.

**A**

**B**

**C**

**Supplementary Figure 2**

Effects of 3 natural compounds on dry weight of *P. paneum* OM1 on YES agar plates after 7 days of incubation at 25°C. **(A)** propyl paraben, **(B)** citral, and **(C)** gallic acid. The mycelial dry weight was measured in triplicate. Data are represented as the mean ± standard deviation. Different letters indicate statistically significant differences (P<0.05).

**A**

**B**

**C**

**D**

**E**

**F**

**G**

**H**

**I**

**Supplementary Figure 3**

HPLC chromatograms of patulin after culture of *P. paneum* OM1 on YES agar plates containing each natural compound or on pears during 6 days at 25°C, and from pear spiked with a patulin standard. **(A)** patulin standard solution (2 μg/mL), **(B)** YES agar plates untreated with any compound, **(C)** YES agar plates containing 10 μg/mL of propyl paraben **(D)** YES agar plates containing 200 μg/mL of citral, **(E)** YES agar plates containing 100 μg/mL of gallic acid, **(F)** pear untreated with propyl paraben, **(G)** pear treated with 0.1 μg of propyl paraben, **(H)** pear unspiked with patulin, **(I)** pear spiked with 1 μg/mL of patulin. The retention time of the patulin peak was 14.4 min.
